# A case of a large solitary fibrous tumour of the uterine cervix

**DOI:** 10.1186/1472-6874-14-3

**Published:** 2014-01-07

**Authors:** Andrzej Nowakowski, Wojciech Kozłowski, Dariusz Włodarczyk, Marta Szajnik-Szczepański, Włodzimierz Baranowski

**Affiliations:** 1 Department of Gynaecology and Oncologic Gynaecology, Military Institute of Medicine, ul. Szaserów 128, 04-141 Warsaw 44, Poland; 2 Department of Pathomorphology, Military Institute of Medicine, ul. Szaserów 128, 04-141 Warsaw 44, Poland

**Keywords:** Cervix, Immunohistochemistry, Solitary fibrous tumour, Surgery

## Abstract

**Background:**

Solitary fibrous tumour of the uterine cervix is an extremely rare phenomenon. We present a case of the largest cervical tumour of this type in this anatomical location reported so far.

**Case presentation:**

A 45-year old white female presented with abdominal pain, abnormal uterine bleedings and a 15 cm mass of the uterine cervix/left parametrium. Histological examination with immunohistochemistry of the tumour biopsy revealed diagnosis of solitary fibrous tumour. The patient underwent radical abdominal hysterectomy with bilateral salpingo-oophorectomy and pelvic lymphadenectomy. No recurrence has been observed for 8 months of follow-up.

**Conclusions:**

Solitary fibrous tumour can be occasionally found in patients with large cervical/parametrial masses. Immunohistochemistry was helpful in diagnosis and surgery was feasible and effective in treatment of our case of a large solitary fibrous tumour of the cervix.

## Background

**Figure 1:**
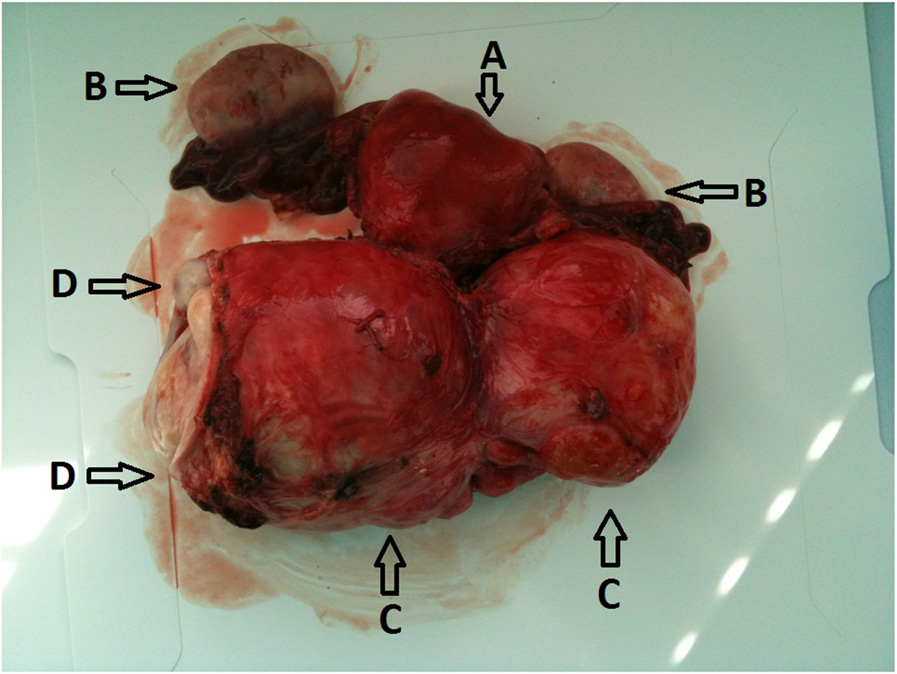
Surgical specimen: uterus body (A), bilateral adnexa (B), two cylindric-shaped parts of the tumour (C) and vaginal cuff (D).

Solitary fibrous tumour (SFT) is a rare mesenchymal neoplasm accounting for less than 2% of all soft-tissue tumours [1]. It may originate at a spectrum of anatomical locations such as the thoracic and abdominal cavities, retroperitoneum and the pelvis [1]. The course of SFT is predominantly benign however 10-15% of tumours may recur and present malignant behaviour [1]. SFTs very rarely arise in female reproductive organs and, to our knowledge, just above ten cases of SFTs were reported in the female reproductive system (vulva, vagina, para-vaginal space, uterus, paraovarian tissue, broad ligament, fallopian tube) [2] and three of them in the uterine cervix [2-4]. We report a case of a SFT of the uterine cervix which grew to a lot larger size than the other three SFTs at this anatomical location described in the literature (Table 1, Figure 1) and was treated effectively by surgery.

**Table 1:** Cases of solitary fibrous tumours of the uterine cervix reported in the literature

**Author**	**Patient’s age (years)**	**Maximal diameter of tumour (cm)**	**Immunohistochemical profile**	**Symptoms**	**Treatment**	**Follow-up**	**Outcome**
Hasegawa *et al*. [3,5]	78	6	(+): MIB1-LI 0.5%, bcl-2, CD34 (-): S100, CK, desmin, CD31, α-SMA, EMA	Abnormal vaginal bleeding	Excision (not specified)	11 years	NR
Sidebotham *et al*. [4]	14	1.7	(+): MIB1-LI 5%, CD34, patchy (+): S100, CD68, focally (+): ER, PR, SMA, (-): bcl-2, desmin, myogenin, WT-1, CD99, CD1a, HMB-45, alk	Abnormal vaginal bleeding	Abdominal radical trachelectomy	2 weeks	Alive, NR
Rahimi *et al*. [2]	68	1.7	(+): vim, CD99, CD34, bcl-2, ER, PR, β-catenin, (-): EMA, S100, factor XIIIa, CKAE1/AE3, caldesmon, desmin, CD31, SMA	None	Robotic assisted radical hysterectomy*	NA	NA
Current case	45	16	(+): CD34, bcl-2, vim, focally (+): SMA, desmin, (-): S100, CKAE1/AE3	Abnormal vaginal bleeding, low abdominal pain	Radical hysterectomy	8 months	Alive, NR

(+) – positive immunostaining, MIB1-LI – MIB1 labeling index, bcl-2 – B-cell lymphoma 2, (-) – negative immunostaining, S100 – S100 protein, CK – cytokeratin, α-SMA – α-smooth muscle actin, EMA - epithelial membrane antigen, NR – no recurrence, ER- estrogen receptor, PR – progesterone receptor, SMA – smooth muscle actin, WT-1 – Wilm’s tumour gene, CD1a – CD1a molecule, HMB-45 – human melanoma black monoclonal antibody, alk – anaplastic lymphoma kinase, vim – vimentin, NA – data not available, CKAE1/AE3 – cytokeratin AE1/AE3, *the solitary fibrous tumour accompanied an invasive cervical cancer. All reported cases had benign histology.

## Case presentation

**Figure 2:**
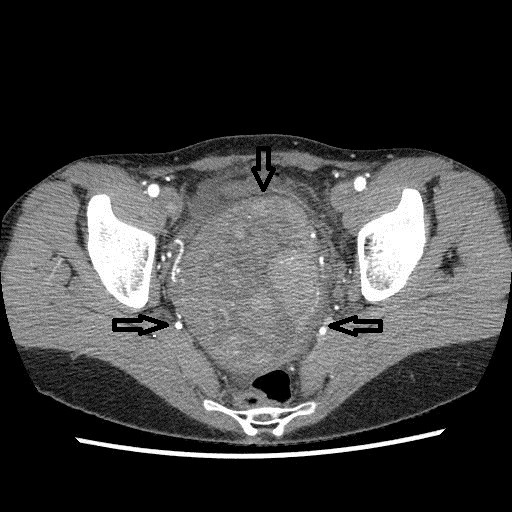
**Axial computed tomography scan of the pelvis.** A large mass arising from the cervix (arrows) and compressing the bladder and the rectum.

**Figure 3:**
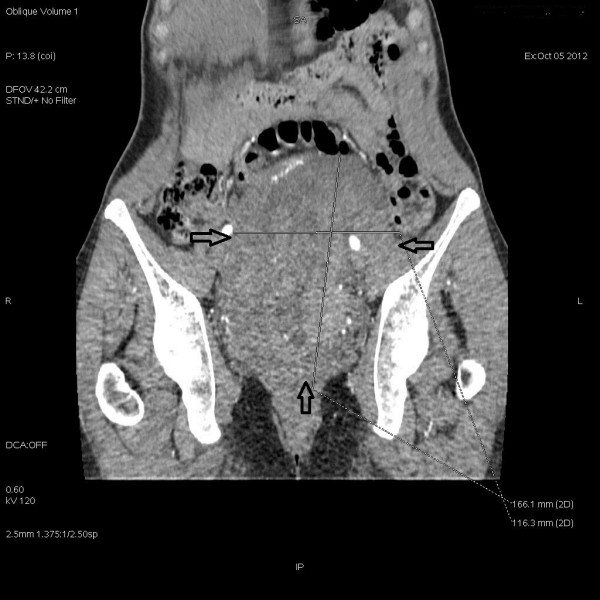
**Coronal computed tomography scan of the pelvis.** A large pathological tumour (arrows) filling up most of the minor pelvis.

**Figure 4:**
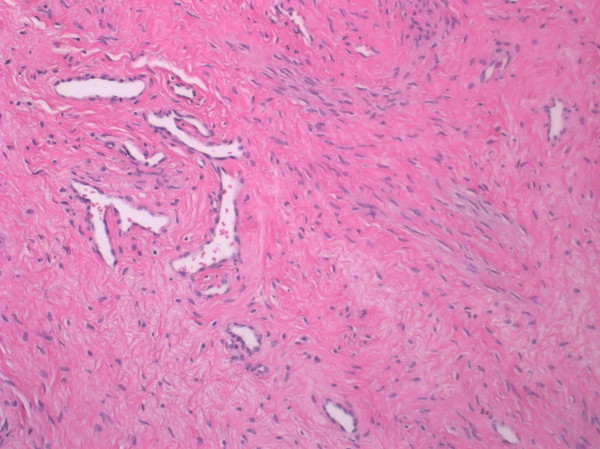
**Microphotograph of the tumour.** Spindle**-**shaped cells within collagenous stroma and prominent medium**-**sized ramified/branching vessels (HE, magnification 200×).

**Figure 5:**
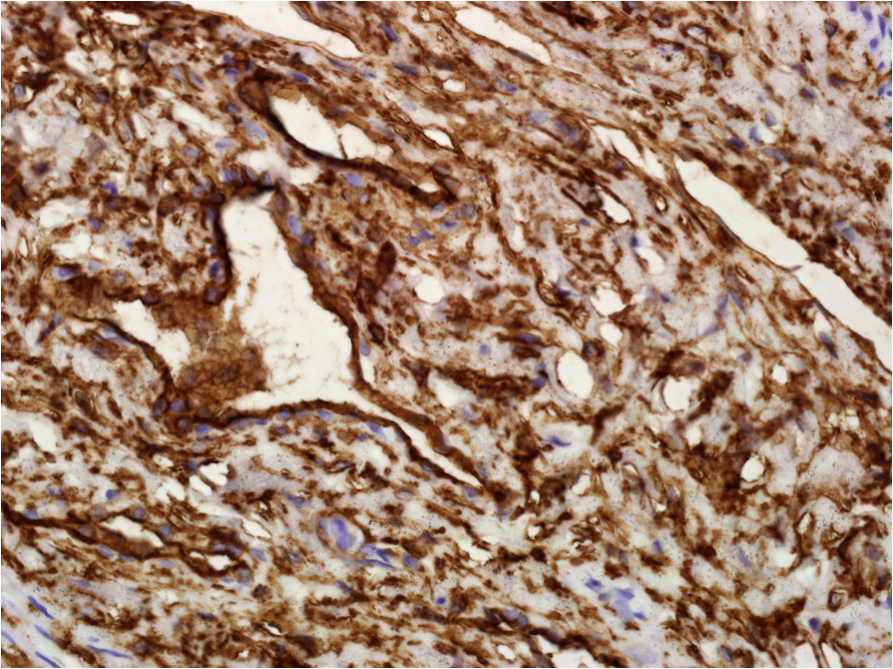
**Microphotograph of the immunohistochemistry specimen.** Strong and diffuse reactivity for CD34 (magnification 400×).

A 45-year-old white women, para 5 aborta 1 presented with lower abdomen pain. She had been treated for misdiagnosed chronic adnexitis for several months at a local clinic. Her obstetric and gynaecological history was significant for one miscarriage, two natural births, one premature breech delivery and two cesarean sections. A year before she had undergone a pelvic examination which did not reveal abnormalities. On speculum and bimanual examination there was a movable tumour of the uterine cervix and left parametrium of approximately 15 cm in size relocating normal size body of the uterus to the right side of the pelvis. Pap test was “negative for intraepithelial lesion or malignancy” and colposcopy revealed the external cervical os relocated to the right vaginal fornix, type 3 transformation zone and normal squamous epithelium covering the vaginal portio and the vaginal fornices. Transvaginal ultrasound showed a 15 cm polycyclic tumour of mixed echogenicity encompassing uterine cervix and isthmus. The uterine body of normal size and shape was relocated to the right side of the pelvis and normal adnexa were visualized. No free fluid was present in the pouch of Douglas. Abdominal and pelvic computed tomography (CT) scan revealed a 160 mm × 109 mm × 98 mm mass in the pelvis (Figures 2 and 3). The mass compressed the uterus to the right, the urinary bladder to the anterior and the rectum to the posterior walls of the pelvis. Numerous extended blood vessels were visualized around the tumour and there was a suspicion of invasion into cervical and vaginal tissues. No other abnormalities in the pelvis and the abdominal cavity were noticed. Chest X-ray and essential blood and urine tests were normal. The patient underwent a diagnostic dilatation and curettage and a wedge-shaped cold knife biopsy of the tumour through the vaginal fornix. Histological examination revealed normal endometrium and endocervix. The tumour biopsy revealed microscopic view of patternless architecture with spindle-shaped tumour cells and collagenous stroma (Figure 4). Immunohistochemistry showed positive staining for CD34 (Figure 5), bcl-2 and vimentin, only focal positivity for smooth muscle actin and desmin and negative staining for S-100, cytokeratin AE1/AE3. The microscopic and immunohistochemical features were consistent with the diagnosis of SFT. The patient was qualified for explorative laparotomy and successfully underwent radical abdominal hysterectomy, bilateral salpingo-oophorectomy and pelvic lympnode dissection. The tumour had a thin capsule and there was no intraoperative signs of metastases or invasion of neighbouring organs. The vasculature around the tumour was rich and anatomical conditions of the pelvis were distorted by the large size and location of the tumour which made the procedure technically difficult. Operating time was 170 minutes, blood loss was 2200 ml and patient received two units of Red Blood Cells, three units of Fresh Frozen Plasma and antibiotic prophylaxis perioperatively. The surgical specimen showed two cylindric parts of the tumour: the first 9.0 cm × 8.5 cm × 7.5 cm and the second 7.5 cm × 6.5 cm × 5.5 cm (Figure 1). The body of uterus was of normal size and shape. Histopathology of the specimen confirmed benign morphology of the tumour and revealed normal adnexa, body of the uterus and lymphnodes. The patient was dismissed from hospital in good condition on the sixth day after surgery. 40 days later she presented with low abdominal pain radiating to the right limb and fever of 38.5° Celsius. A tumour suspicious for abscess or hematoma of 28 mm × 36 mm was localized by the right wall of the minor pelvis in a CT scan. The symptoms and the tumour resolved within two weeks after antibiotic treatment and the follow-up has been uneventful with no signs of recurrence for 8 months now.

## Conclusions

Rare histotypes of tumours such as solitary fibrous tumour can be occasionally found in patients with large cervical/parametrial masses and may cause abnormal uterine bleedings (Table 1).

Our case and all the other three described SFTs of the cervix had benign histology (Table 1). In one case, the SFT described as a polyp in the cervical canal, accompanied stage FIGO Ib1 squamous cervical cancer [2].

Although there is no clearly defined morphologic and immunoreactivity criteria of SFT and the four described SFTs of the cervix differ somewhat in the immunoprofile (Table 1), immunohistochemical detection of CD34 and bcl-2 was helpful in our preoperational diagnosis of the large biopsy specimen, since the two markers were suggested to differentiate SFT from other spindle-cell tumours [3].

Surgery was feasible and has been effective so far in the very large cervical SFT we treated, similarly as in the other two women with cervical SFTs for which follow-up data are available (Table 1). The experience with the post-treatment course of cervical SFTs is therefore scarce. Follow-up is required since recurrences and malignant course were reported for these types of tumours in other anatomical locations [1].

## Consent

Written informed consent was obtained from the patient for publication of this case report and any accompanying images. A copy of the written consent is available for review by the Editor of this journal.

## Abbreviations

SFT: Solitary fibrous tumour; CT: Computed tomography.

## Competing interests

The authors declare that they have no competing interests.

## Authors’ contributions

AN took a major part in the diagnosis, treatment and follow-up of the patient and planned, wrote and reviewed the manuscript. WK provided histopathological work-up and description of the histological diagnosis. DW was involved in surgical treatment of the patient, provided selection of the CT scans. MSS took part in conception of the manuscript, literature search and review. WB supervised the treatment of the patient, took part in the conception of the manuscript. All authors critically reviewed and approved the manuscript.

## Pre-publication history

The pre-publication history for this paper can be accessed here:

http://www.biomedcentral.com/1472-6874/14/3/prepub
